# Supplementary material for: Mouse models of pediatric high-grade gliomas with MYCN amplification reveal intratumoral heterogeneity and lineage signatures
Source: Nat Commun. 2023 Nov 24;14:7717. doi: 10.1038/s41467-023-43564-w (PMC10673884; doi:10.1038/s41467-023-43564-w)
Supplement: Supplementary file 3 — Description of Additional Supplementary Files [file 41467_2023_43564_MOESM3_ESM.pdf]

Description of Additional Supplementary Files

Supplementary data 1: AUC values for all substances in HTDS screen for mouse HGG-MYCN cell line (PN003) and human cell line (pbt04). The AUC was determined after incubating the indicated cell lines with different concentrations of the inhibitors by a Cell Titer Glo viability assay. All values were determined in triplicates. A high AUC corresponds to a low sensitivity and vice versa.
