# Supplementary material for: Mouse models of pediatric high-grade gliomas with MYCN amplification reveal intratumoral heterogeneity and lineage signatures
Source: Nat Commun. 2023 Nov 24;14:7717. doi: 10.1038/s41467-023-43564-w (PMC10673884; doi:10.1038/s41467-023-43564-w)
Supplement: Supplementary file 5 — Reporting Summary [file 41467_2023_43564_MOESM5_ESM.pdf]

nature portfolio

Corresponding author(s): Ulrich Schüller

Last updated by author(s): Oct 23, 2023

Reporting Summary

Nature Portfolio wishes to improve the reproducibility of the work that we publish. This form provides structure for consistency and transparency in reporting. For further information on Nature Portfolio policies, see our Editorial Policies and the Editorial Policy Checklist.

Statistics

For all statistical analyses, confirm that the following items are present in the figure legend, table legend, main text, or Methods section.

n/a	Confirmed	
☐	☒	The exact sample size (*n*) for each experimental group/condition, given as a discrete number and unit of measurement
☐	☒	A statement on whether measurements were taken from distinct samples or whether the same sample was measured repeatedly
☐	☒	The statistical test(s) used AND whether they are one- or two-sided<br>*Only common tests should be described solely by name; describe more complex techniques in the Methods section.*
☐	☒	A description of all covariates tested
☐	☒	A description of any assumptions or corrections, such as tests of normality and adjustment for multiple comparisons
☐	☒	A full description of the statistical parameters including central tendency (e.g. means) or other basic estimates (e.g. regression coefficient) AND variation (e.g. standard deviation) or associated estimates of uncertainty (e.g. confidence intervals)
☐	☒	For null hypothesis testing, the test statistic (e.g. *F*, *t*, *r*) with confidence intervals, effect sizes, degrees of freedom and *P* value noted<br>*Give P values as exact values whenever suitable.*
☒	☐	For Bayesian analysis, information on the choice of priors and Markov chain Monte Carlo settings
☐	☒	For hierarchical and complex designs, identification of the appropriate level for tests and full reporting of outcomes
☒	☐	Estimates of effect sizes (e.g. Cohen's *d*, Pearson's *r*), indicating how they were calculated

Our web collection on statistics for biologists contains articles on many of the points above.

Software and code

Policy information about availability of computer code

Data collection	RNA Seq.: Raw fastq files of mouse samples were processed in usegalaxy.eu42. Low quality reads were detected using FastQC (Galaxy Version 0.73+galaxy0). Trimmomatic (Galaxy Version 0.38.1) was used for trimming poor quality reads (reads with average quality < 20). Reads were aligned to the mm39.ncbiRefseq.gtf.gz using STAR aligner (Galaxy Version 2.7.8a+galaxy2) and gene expression was quantified using featureCounts (Galaxy Version 2.0.1+galaxy1).<br>ScRNA Seq: The samples were analyzed with the 10x Genomics CellRanger v6.0.2 pipeline54 and Seurat R package v4.0.555. Raw data was converted to fastq format with the CellRanger mkfastq function and then aligned against the murine reference transcriptome mm10 v2020-A with CellRanger count and default values. Seurat objects were generated for the samples based on the following filter criteria: at least three cells, a minimum feature count of 200, and cells with < 25% of mitochondrial genes<br>DNA Methylation: Raw idat files of mouse DNA methylation arrays generated with the iScan device were processed with the openSeSAMe pipeline (v1.12.9).
Data analysis	RNA Seq.: DEseq2(Galaxy Version 2.11.40.7+galaxy2) was used for generating VST-normalized files for all samples. Mouse samples were measured in two different batches, and the VST- normalized files were combined and corrected for batch effect using ComBat from sva package (3.44.0) in Rstudio (4.2.1). Average tumor subgroup-specific gene expression was used for calculating euclidean distance. Sample-sample distance plot was visualized using ComplexHeatmap (2.12.1). Limma (3.52.2) was used for performing differential expression analysis. Top 500 differentially expressed genes (adjusted for multiple testing using Bonferroni-Hochberg correction and sorted by F-statistic) were visualized using umap (0.2.9.0) and ComplexHeatmap (2.12.1) in Rstudio. AGDEX was performed in C++ as described previously. Gene set enrichment analysis (GSEA) was performed using the software GSEA v4.2.0 of the Broad Institute.<br>DNA Methylation: Human data was processed with minfi and noob-normalized beta-values were employed for cluserting. Mouse data were processed with SeSAMe (v1.12.9), beta values were extracted and the data were quantile normalized. The conumee package (v1.28.0) was

used for generating CNV data. CNV values of the bin signals were calculated and depicted as a heatmap using ComplexHeatmap
ScRNASeq.: The filtered data was normalized, integrated, and clustered with Seurat, using a resolution parameter of 0.5. Feature plots, UMAPs, dotplots and heatmap visualizations were created with Seurat functions. Lists of differentially expressed genes per cluster were calculated with Seurat's findMarkers function, using the MAST test "hinter" using a resolution parameter of 0.5. Finally, a logistic regression analysis was used to identify similarities between the murine scRNA-seq clusters and reference datasets. Briefly, a multinomial regression model was trained on the chosen reference dataset with the R package glmnet, and cell types were predicted for the original mouse data based on this model. Probabilities per reference cell type were aggregated on cluster level using R's mean function, and visualized as heatmaps with the package pheatmap.

For manuscripts utilizing custom algorithms or software that are central to the research but not yet described in published literature, software must be made available to editors and reviewers. We strongly encourage code deposition in a community repository (e.g. GitHub). See the Nature Portfolio guidelines for submitting code & software for further information.

Data

Policy information about availability of data

All manuscripts must include a data availability statement. This statement should provide the following information, where applicable:

- Accession codes, unique identifiers, or web links for publicly available datasets
- A description of any restrictions on data availability
- For clinical datasets or third party data, please ensure that the statement adheres to our policy

The DNA methylation and RNA sequencing data including the raw data generated in this study have been deposited in the GEO database under accession code GSE227413 [https://www.ncbi.nlm.nih.gov/geo/query/acc.cgi?acc=GSE227413]. The scRNA sequencing data has been deposited in the GEO database under accession code GSE237237 [https://www.ncbi.nlm.nih.gov/geo/query/acc.cgi?acc=GSE237237].
The human gene expression data was obtained from GSE73038 [https://www.ncbi.nlm.nih.gov/geo/query/acc.cgi?acc=GSE73038]. Mouse scRNA sequencing data publicly available from GSE134918 [https://www.ncbi.nlm.nih.gov/geo/query/acc.cgi?acc=GSE134918] was employed in this study. AUC data from other glioma cell lines were provided by Marc Remke and Nan Qin. Source data are provided with this paper.

Human research participants

Policy information about studies involving human research participants and Sex and Gender in Research.

Reporting on sex and gender	We included brain tumor samples of 2 male and 3 female patients. We only used anonymized data and deducted the sex from the methylation data without any sex selection.
Population characteristics	Population characteristics our data mostly includes primary biopsies from tumors in children (under 18 years old) with no selection on a specific sex.
Recruitment	No participants were recruited to this study. We used publically available data on patient collections where a specific brain tumor was identified upon diagnosis. Apart from that we used biopsy specimen from the University Medical Center Hamburg-Eppendorf.
Ethics oversight	We used publically available data on patient collections where a specific brain tumor was identified.The use of biopsy-specimens for research upon anonymization was always in accordance with local ethical standards and regulations at the University Medical Center Hamburg-Eppendorf

Note that full information on the approval of the study protocol must also be provided in the manuscript.

Field-specific reporting

Please select the one below that is the best fit for your research. If you are not sure, read the appropriate sections before making your selection.

☒ Life sciences ☐ Behavioural & social sciences ☐ Ecological, evolutionary & environmental sciences

For a reference copy of the document with all sections, see nature.com/documents/nr-reporting-summary-flat.pdf

Life sciences study design

All studies must disclose on these points even when the disclosure is negative.

Sample size	Sample sizes for in vivo experiments were not statistically determined but set on the basis of agreeing to a size suitable for ethical regulations while also large enough to ensure strong statistical power. Sample sizes were large enough to ensure results obtained were of a representable quantity and quality. For global gene expression analysis more than 3 samples of each group were included.<br>For in vitro experiments, initial samples size for the number of new cell lines generated was n=6, which was determined comparable to the sample sizes for other in vivo experiments. For high-throughput drug-screening, a representative cell line was directly compared against another representative line. Such experiments were always confirmed and repeated at least three times. When histological analyses were performed, numerous biological repeats were conducted to ensure a representative overview. Here no statistical method was used to determine the sample size as a representative average should be obtained.

Data exclusions	No data was excluded.
Replication	In vitro experiments were replicated numerous times (>3). Analysis of animal tissue was carried out for many animals across multiple generations to ensure no genetic changes occured and that all data shown was an accurate and average representation of these samples. Where appropriate, statistical analysis has been carried out for these replicates. The work we present is reliable as it is a combination of many findings, both confirmed in vivo and in vitro.
Randomization	No randomization was performed as no groups were assigned which would need to be randomized.
Blinding	Blinding was performed where the responsible researcher blinded results that were analyzed/quantified. In vivo work and mouse symptoms were assessed by multiple researchers as well as the technical staff in the animal facility. Unless stated otherwise, all mice were sacrificed at a humane endpoint judged to be similar across all individual animals.

Reporting for specific materials, systems and methods

We require information from authors about some types of materials, experimental systems and methods used in many studies. Here, indicate whether each material, system or method listed is relevant to your study. If you are not sure if a list item applies to your research, read the appropriate section before selecting a response.

Materials & experimental systems

n/a	Involved in the study	
☐	☒	Antibodies
☐	☒	Eukaryotic cell lines
☒	☐	Palaeontology and archaeology
☐	☒	Animals and other organisms
☒	☐	Clinical data
☒	☐	Dual use research of concern

Methods

n/a	Involved in the study	
☒	☐	ChIP-seq
☒	☐	Flow cytometry
☒	☐	MRI-based neuroimaging

Antibodies

Antibodies used	MYCN (Cell Signaling, 517053, 1:1000), P53 (Dako, M7001, 1:800), Ki67 (Abcam, ab16667, 1:100), Nestin (Abcam, ab221660, 1:2000), SOX2 (Abcam, ab97959, 1:200), OLIG2 (Millipore, AB9610, 1:200), Cre (Covance / DCS-diagnostics, PRB-106P, 1:100) and GFAP (Dako, M0761, 1:200).
Validation	Antibodies were used as per the manufacturers' instructions. The initial validation was performed by the manufacturers. All stainings included a positive and a negative control (within the same tissue or cre-negative control animals) to ensure technically successful stainings.

Eukaryotic cell lines

Policy information about cell lines and Sex and Gender in Research

Cell line source(s)	The pn003 cell line were derived from tumors of the transgenic mice generated in this study.<br>Pbt-04 cells were received from SCRI, Brain tumor resource lab.
Authentication	Pbt-04 cells were authenticated by their Copy Number profile generated from methylation array data.
Mycoplasma contamination	All cell lines were tested negatively from mycoplasma contamination by using a Mycoplasma detection kit (PCR Mycoplasma Test Kit I/C) from PromoKine.
Commonly misidentified lines (See ICLAC register)	No commonly misidentified cell lines were employed in the study.

Animals and other research organisms

Policy information about studies involving animals; ARRIVE guidelines recommended for reporting animal research, and Sex and Gender in Research

Laboratory animals	We used previously described mouse lines: hGFAP-cre (JAX:4600), Blbp-cre (MGI:3810647), Sox2-cre (JAX:008454), Trp53Fl/Fl (JAX:008462) and lsl-MYCN (MGI:6196127) as well as Math1-cre::SmoM2Fl/wt (doi: 10.1016/j.ccr.2008.07.005.) transgenic mouse lines and generated a new transgenic mouse strain crossed from the aforementioned mice.<br>We used mice aged 8-30 weeks for mating, hGFAP-cre::lsl-MYCN::TP53Fl/Fl mice became symptomatic with 80-90 days and were sacrificed, Math1-cre::SmoM2 Fl/Fl mice were sacrificed at P12.
Wild animals	No wild animals were used.

Reporting on sex	In transgenic animals both female and male mice develop tumors spontaneously (no significant differences).
Field-collected samples	This study did not include field-collected samples.
Ethics oversight	All animal procedures were performed in accordance with applicable animal protection laws and approved by the state of Hamburg (Reference N2019/99)

Note that full information on the approval of the study protocol must also be provided in the manuscript.
