# Supplementary material for: Mouse models of pediatric high-grade gliomas with MYCN amplification reveal intratumoral heterogeneity and lineage signatures
Source: Nat Commun. 2023 Nov 24;14:7717. doi: 10.1038/s41467-023-43564-w (PMC10673884; doi:10.1038/s41467-023-43564-w)
Supplement: Supplementary file 1 — Supplementary Information [file 41467_2023_43564_MOESM1_ESM.pdf]

***Supplementary table 1*: Primer pairs for genotyping**

**Target**	**Forward**	**Reverse**
Cre	TCCGGGCTGCCACGACCAA	GGCGCGGCAACACCATTTT
MYCN floxed allele	ACCACAAGGCCCTCAGTACC	TGGGACGCACAGTGATGG
MYCN wildtype allele	CTCTTCCCTCGTGATCTGCAACTCC	CATGTCTTTAATCTACCTCGATGG
MYCN recombined allele	GCCCGCGGTGATGCCTTTGAGG	CGGGGACTGGGCGGTGGAAC
TP53	CACAAAAAACAGGTTAAACCCAGC	GCACCTTTGATCCCAGCACATA
TP53 recombined allele	CACAAAAAACAGGTTAAACCCAGC	GAAGACAGAAAAGGGGAGGG
SmoM2 floxed allele	AAGTTCATCTGCACCACCG	TGCTCAGGTAGTGGTTGTCG
SmoM2 wildtype allele	GGAGCGGGAGAAATGGATATG	CGTGATCTGCAACTCCAGTC

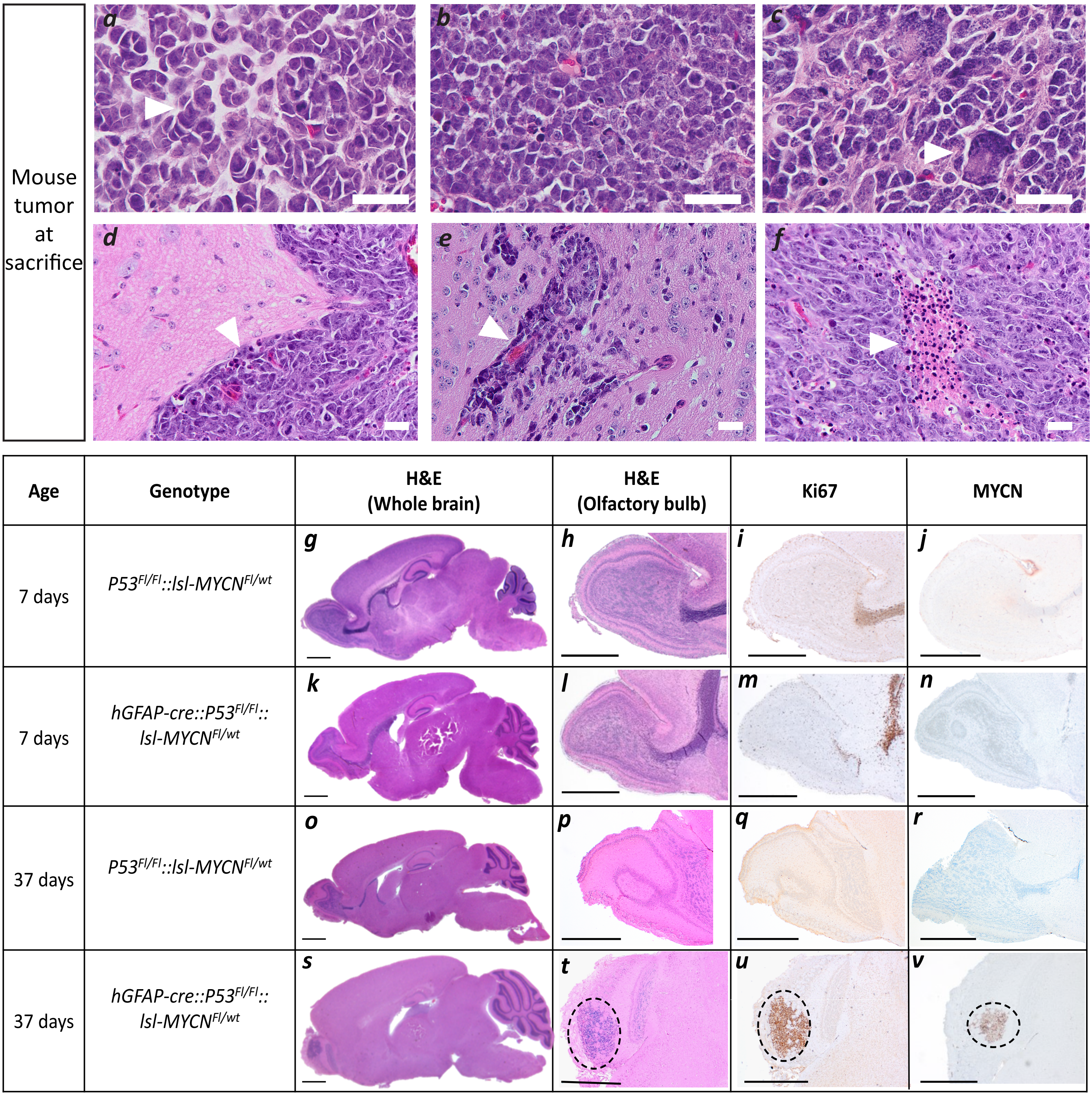

***Figure S1*: Murine HGG-MYCN show a high intra-tumoral heterogeneity in cell histology, mostly clear demarcation and a high frequency of necrosis. Tumors develop postnatally between day 7 and 37.** Cell histology of murine HGG-MYCN shows different cell growth patters including distinct cell clusters (a) but densely packed nuceli none or only little cytoplasm visible (b). Giant cells can also be observed in mHGG-MYCN (c, arrow). (d) The tumors are clearly demarcated from the normal tissue. (e) Rarely, tumor cell clusters are infiltrating the brain parenchyma. (f) In the majority of tumors (6/10), necrosis of different sizes are detected. (g-v) Representative histological pictures of 7- and 37-day-old mice. At day seven, no tumor lesions were detected, whereas at day 37, strongly proliferating lesions were detectable in the olfactory bulb (circle). The scale bar in (a-c) correspond to 20 µm, the scalebar in (d-f) to 20 µm. Scale bars in (g, k, o and s) correspond to 1 mm, and the scale bar in (h-j), (l-n), (p-r) and (t-v) corresponds to 1 mm.

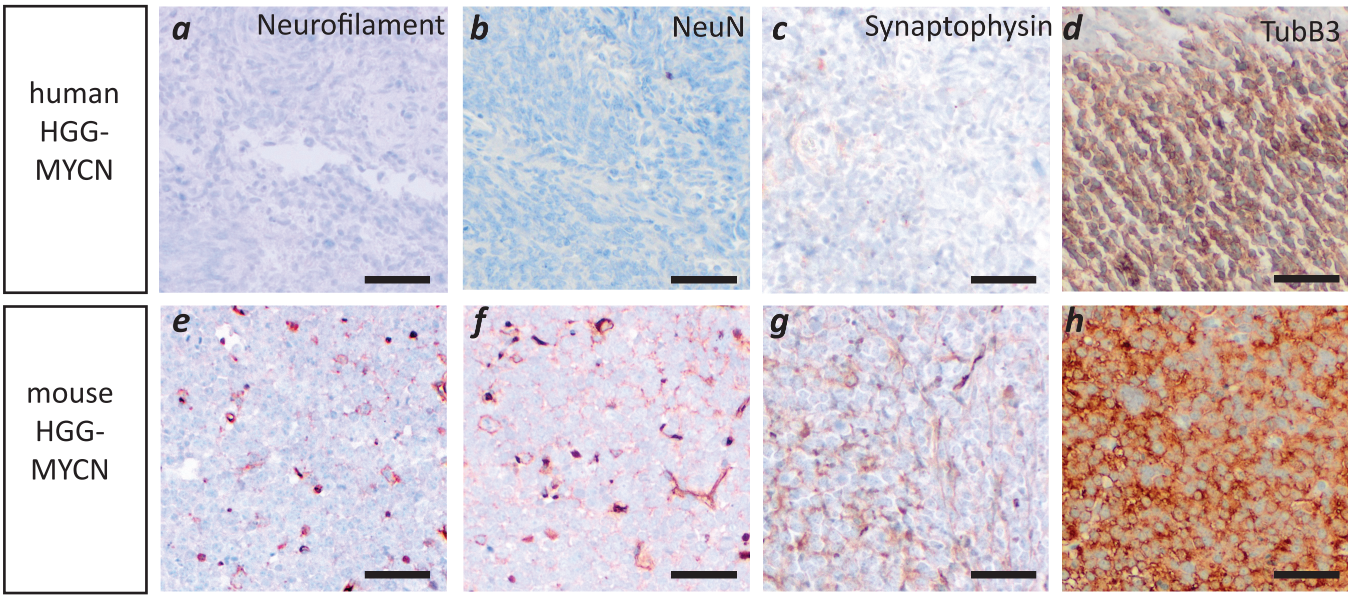

***Figure S2*: Human and murine HGG-MYCN show similar marker expression.** Tumors of neither species express Neurofilament (a,e), NeuN (b,f) or Synaptophysin(c,g) but express ß-3-Tubulin (TubB3) (d,h). The scale bar correponds to 50 µm in (a-h).

a

Age	Litters	Genotype	Counted	Expected frequency	Counted frequency
E16.5	2	*Trp53*$^{Fl/Fl}$*::lsl-MYCN*$^{Fl/wt}$	11	50%	64%
		Sox2-cre::Trp53$^{Fl/wt}$*::lsl-MYCN*$^{Fl/wt}$	1	25%	6%
		Sox2-cre::Trp53$^{Fl/Fl}$*::lsl-MYCN*$^{Fl/wt}$	5	25%	30%
P0	7	*Trp53*$^{Fl/Fl}$*::lsl-MYCN*$^{Fl/wt}$	27	50%	100%
		Sox2-cre::Trp53$^{Fl/wt}$*::lsl-MYCN*$^{Fl/wt}$	0	25%	0%
		Sox2-cre::Trp53$^{Fl/Fl}$*::lsl-MYCN*$^{Fl/wt}$	0	25%	0%

	Trp53$^{Fl/Fl}$***::lsl-MYCN***$^{Fl/wt}$	***Sox2-cre::Trp53***$^{Fl/Fl}$***::lsl-MYCN***$^{Fl/wt}$
**E16.5**	b (image)	c, d (images)

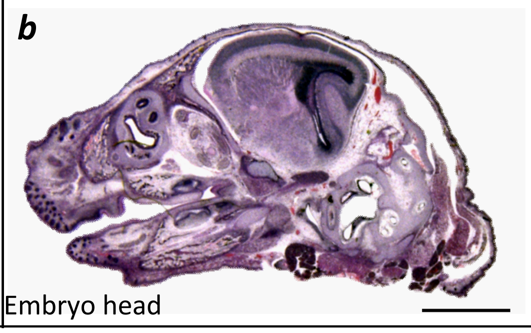

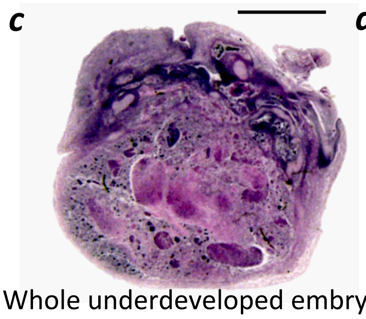

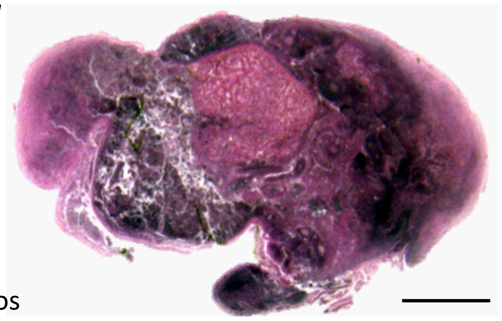

e

Litters	Genotype	Counted	Expected frequency	Counted frequency
10	*Trp53*$^{Fl/Fl}$*::lsl-MYCN*$^{Fl/wt}$	51	50%	82%
	Blbp-cre::Trp53$^{Fl/wt}$*::lsl-MYCN*$^{Fl/wt}$	7	25%	11%
	Blbp-cre::Trp53$^{Fl/Fl}$*::lsl-MYCN*$^{Fl/wt}$	2	25%	3%
	Blbp-cre::Trp53$^{Fl/Fl}$*::lsl-MYCN*$^{Fl/Fl}$	2	25%	3%

Blbp-cre::Trp53$^{Fl/wt}$***::*** ***lsl-MYCN***$^{Fl/wt}$	***Blbp-cre::Trp53***$^{Fl/Fl}$***::*** ***lsl-MYCN***$^{Fl/wt}$	***Blbp-cre::Trp53***$^{Fl/Fl}$***::*** ***lsl-MYCN***$^{Fl/Fl}$
f (image)	g (image)	h (image)

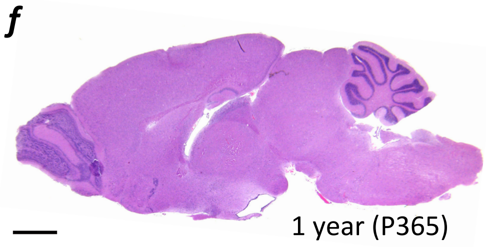

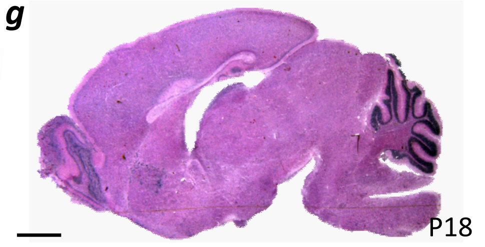

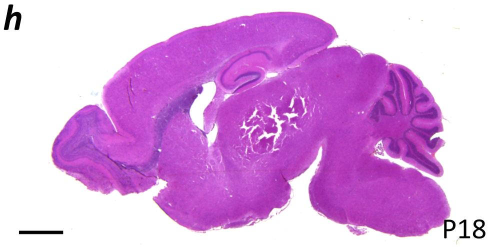

Figure S3: **Mouse HGG-MYCN do not develop from Sox2- or Blbp-derived cell populations in the developing brain. (a)** *Sox2-cre::Trp53$^{Fl/Fl}$::lsl-MYCN* mice were bred. Although 25% of the offspring should carry the transgenes, no animal with the expected genotype was born, indicating embryonic lethality of the genotype. (b) Histological image of a control embryo head at E16.5. (c) Image of an extremely underdeveloped *Sox2-cre::Trp53$^{Fl/Fl}$::lsl-MYCN* animal at E16.5. No organs or other structures were identified. (d) A second highly underdeveloped *Sox2-cre::Trp53$^{Fl/Fl}$::lsl-MYCN* animal at E16.5, in which different body structures were visible, but could not be clearly assigned to organs. (e) Targeting the developing CNS under the Blbp promoter was embryonically lethal. Only four animals carrying at least one MYCN allele and a homozygously floxed Trp53-allele survived until birth. Two of them were sacrificed at P0 due to neuro-developmental symptoms and the other two developed a hydrocephalus and were sacrificed at day 18. (f) H&E image of a *Blbp-cre::Trp53$^{Fl/wt}$::lsl-MYCN$^{Fl/wt}$* mouse brain without abnormalities. The brains of a *Blbp-cre::Trp53$^{Fl/Fl}$::lsl-MYCN$^{Fl/wt}$* (g) and a *Blbp-cre::Trp53$^{Fl/Fl}$::lsl-MYCN$^{Fl/Fl}$* mouse (h) showed slightly enlarged ventricles, but no other abnormalities or signs of tumor development. Scale bar in b-d corresponds to 2 mm, and the scale bar in f-h corresponds to 2 mm.

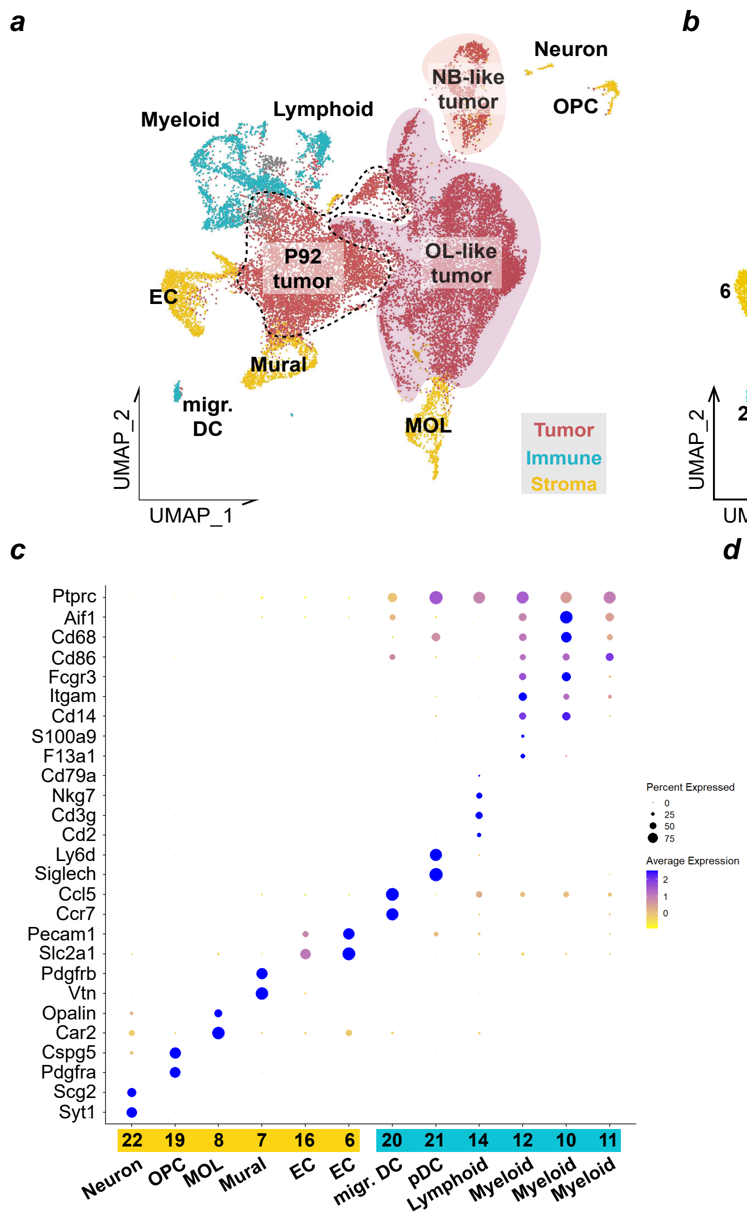

d

AC	Gfap	OL-like	Plp1
	Aqp4		Mbp
	Clu		Cnp
	F3		Ptgds
	Mlc1		Dbi
	Agt		Ckb
	Hopx		Cryab
	Pltp		Arpc1a
	Ramp1		Sirt2
	Ednrb		Cldn11
MES	Adm	NB-like	Stmn2
	Angptl4		Sox11
	Epas1		Rtn1
	Egln3		Neurod1
	Anxa1		Basp1
	Serpine1		Casp3
	Igfbp3		Ina
	Tnfrsf1a		Gap43
	Plod2		Dcx
	Ddit3		Rbfox3
OPC	Ptprz1		
	Pdgfra		
	Cspg5		
	Lhfpl3		

Figure S4:* Signature genes defining cell clusters of scRNA sequencing analysis. *(a-b) UMAP of scRNA seq data including all tumor cells. Color coded are tumor cells, and TME, consisting of Immune and stroma cells. The TME cells can be clearly assigned to specific cell classes based on the genes depicted in (c). In order to define different tumor cell clusters, specific marker genes were employed depicted in (d).
